# Single-Step qPCR and dPCR Detection of Diverse CRISPR-Cas9 Gene Editing Events *in Vivo*

**DOI:** 10.1534/g3.117.300123

**Published:** 2017-08-31

**Authors:** Micol Falabella, Linqing Sun, Justin Barr, Andressa Z. Pena, Erin E. Kershaw, Sebastien Gingras, Elena A. Goncharova, Brett A. Kaufman

**Affiliations:** * University of Pittsburgh School of Medicine, Department of Medicine, Division of Cardiology, Center for Metabolism and Mitochondrial Medicine and Vascular Medicine Institute, Pennsylvania 15261; † Integrated DNA Technologies, Coralville, Iowa 52241; ‡ University of Pittsburgh School of Medicine, Department of Medicine, Vascular Medicine Institute, Pennsylvania 15261; § University of Pittsburgh School of Medicine, Department of Medicine, Division of Endocrinology, Pennsylvania 15261; ** University of Pittsburgh School of Medicine, Department of Immunology, Pennsylvania 15261; †† University of Pittsburgh School of Medicine, Department of Medicine, Division of Pulmonary, Allergy and Critical Care Medicine, Vascular Medicine Institute, and Department of Bioengineering, Pennsylvania 15261

## Abstract

Clustered Regularly Interspaced Short Palindromic Repeats (CRISPR)-CRISPR-associated protein 9 (Cas9)-based technology is currently the most flexible means to create targeted mutations by recombination or indel mutations by nonhomologous end joining. During mouse transgenesis, recombinant and indel alleles are often pursued simultaneously. Multiple alleles can be formed in each animal to create significant genetic complexity that complicates the CRISPR-Cas9 approach and analysis. Currently, there are no rapid methods to measure the extent of on-site editing with broad mutation sensitivity. In this study, we demonstrate the allelic diversity arising from targeted CRISPR editing in founder mice. Using this DNA sample collection, we validated specific quantitative and digital PCR methods (qPCR and dPCR, respectively) for measuring the frequency of on-target editing in founder mice. We found that locked nucleic acid (LNA) probes combined with an internal reference probe (Drop-Off Assay) provide accurate measurements of editing rates. The Drop-Off LNA Assay also detected on-target CRISPR-Cas9 gene editing in blastocysts with a sensitivity comparable to PCR-clone sequencing. Lastly, we demonstrate that the allele-specific LNA probes used in qPCR competitor assays can accurately detect recombinant mutations in founder mice. In summary, we show that LNA-based qPCR and dPCR assays provide a rapid method for quantifying the extent of on-target genome editing *in vivo*, testing RNA guides, and detecting recombinant mutations.

CRISPR-Cas9 is a revolutionary genome editing tool derived from the bacterial adaptive immune system (Jinek *et al.* 2012; Mali *et al.* 2013; Sternberg and Doudna 2015). In the past few years, CRISPR-Cas9 technology has been used in numerous biomedical applications, including identification of the molecular basis of genetic disorders (Heidenreich and Zhang 2016), the causes of drug resistance (Chen *et al.* 2015; Ruiz *et al.* 2016), and in the development of new therapeutic strategies (Wang *et al.* 2013; Cox *et al.* 2015). The CRISPR-Cas9 system relies on the ability of the Cas9 endonuclease, directed by a single guide RNA (sgRNA) to the complementary DNA target site, to induce a site-specific double-strand break (DSB). The DNA damage generated by Cas9 can be then repaired by two different mechanisms: homology-directed repair (HDR) or nonhomologous end-joining (NHEJ) (Heidenreich and Zhang 2016). For genome editing, the HDR mechanism enables site-directed mutagenesis near the DSB site through recombination with a donor template, whereas the error-prone NHEJ repair mechanism leads to the formation of indels at the site of cleavage. Due to its ease of targeting, CRISPR-Cas9 enables the rapid generation of mutations, leading to an explosion of novel mouse models, which has consequently created a need for rapid, easy detection of *in vivo* editing in founder animals.

It is increasingly recognized that multiple alleles can be generated in a single founder mouse (Oliver *et al.* 2015; Zhang *et al.* 2016). Engineered mutations created by donor DNA recombination include a restriction-based strategy for genotyping, whereas indel mutations are commonly genotyped by direct Sanger sequencing (Dehairs *et al.* 2016) and, once identified, by probe competition assay (Zentilin and Giacca 2007) or by singleplex qPCR strategies (Yu *et al.* 2014). Direct Sanger sequencing is nonquantitative and relies on high-quality sequence to detect up to three alleles; however, more alleles can be generated in a founder animal, causing the least prevalent sequences to be missed by this approach. PCR-clone sequencing increases the number of variants detected, but the precision of quantitation is limited by the number of clones sequenced and requires significant time and labor. CRISPR-Cas9 editing generates diverse NHEJ alleles, making probe competition assay development for all possible variants impractical. Several recent techniques assume a range of sequence variations (Kc *et al.* 2016) or size differences (Yang *et al.* 2015). These prescriptive approaches do not embrace the reality of broad allele composition in founder animals, where many of these alleles are useful for gene disruption. Rapid detection of undefined editing events at CRISPR-Cas9 target sites is crucial to expedient mouse colony management. Singleplex qPCR has been employed to detect sequence deviation at the CRISPR-Cas9 cut site in zebrafish (Yu *et al.* 2014) and served as the starting point for this study. In this work, we show the genetic complexity that derives from CRISPR-Cas9 genome editing in mouse. Importantly, we demonstrate significant oligo probe insensitivity to sequence mismatching, preventing accurate detection of CRISPR editing. To resolve this limitation, we developed qPCR and dPCR methods that increase editing detection sensitivity to enable the quantification of total CRISPR-mediated editing or to detect HDR alleles. The approaches developed are rapid and applicable to sgRNA testing *in vivo*, founder mice characterization, and colony genotyping.

## Materials and Methods

### Production of sgRNA and generation of T1079A, T1079D LATS1, and R457Q CREBRF mice

The methods for the targeting strategy and the generation of CRISPR-Cas9 edited mice by recombination-mediated mutagenesis have been described (Pelletier *et al.* 2015). Briefly, for the synthesis of the sgRNA, a double-stranded linear DNA template was created by annealing a target-specific primer (TSP) with a common primer containing the full tracrRNA sequence (sgRNA-Scaffold-Primer) and then PCR amplified (Bassett and Liu 2014). The TSP contains a T7 promoter, the target sequence [without protospacer-adjacent motif (PAM)], and part of the tracrRNA sequence, while the common primer contains the full tracrRNA sequence (sgRNA-Scaffold-Primer). The purified PCR product was used as template for the synthesis of the sgRNA using MEGAshortscript T7 kit (ThermoFisher Scientific). The Cas9 mRNA was produced using a linearized plasmid as a template for the *in vitro* transcription mMESSAGE mMACHINE T7 Ultra kit (ThermoFisher Scientific) as previously described (Pelletier *et al.* 2015). Both sgRNA and Cas9 mRNA were purified using a MEGAclear kit (ThermoFisher Scientific) following the manufacturer’s instructions, and then RNA integrity was confirmed using a Bioanalyzer or Tape station (Agilent Technologies).

C57BL/6J pronuclear-stage zygotes, obtained by natural mating of superovulated females, were microinjected with guide RNA (10 ng/μl), Cas9 mRNA (20 ng/μl), and with (mouse production) or without (sgRNA testing *ex vivo*) single-stranded oligodeoxynucleotide (ssODN) Ultramer (0.2 pmol/μl). Injected zygotes were cultured either overnight and transferred to pseudopregnant CD1 receipted females to obtained founder mice, or cultured for 4 d to the blastocysts stage and then harvested.

### Design of drop-off probes and primers

The murine Large Tumor Suppressor Kinase 1 (LATS1) Oligo and LNA Target Probes were designed to detect the editing events at the Cas9 cleavage site, using three consecutive LNA bases in the probe to destabilize binding to non-wt sequences. The wt and R457Q LNA Target probes for the CREBRF genotyping assay were designed to have two consecutive locked bases on the R457Q mutation site, to improve their hybridization specificity to the target sequence. Thermodynamics and hybridization profiles for all probes were determined using the Biophysics tool from Integrated DNA Technologies (IDT; Coralville, IA: biophysics.idtdna.com) with default settings adjusted to match standard qPCR experimental conditions (Mg^2+^ = 3.0 mM and dNTPs = 0.8 mM). To ensure the probes bind to the template prior to the DNA extension, the LNA Target and Reference Probes were designed with similar melting temperatures, both 3–6° higher than the T_m_ of the primers.

### PCR and cloning of individual allelic variants

Total DNA was extracted from the toe/tail biopsy from 20 LATS1 and 6 CREBRF founder mice as described (Kolesar *et al.* 2013) and amplified with Taq DNA Polymerase (New England Biolabs) using the primer sets reported in Supplemental Material, Table S1. The PCR was performed in a ProFlex thermal cycler (ThermoFisher Scientific) with the following PCR amplification profile: one cycle of 94° for 2 min; 35 cycles of 94° for 20 sec, 55° for 20 sec, and 68° for 60 sec; and one cycle of 68° for 5 min. The PCR products were cloned using the TOPO 4 kit with *Escherichia coli* DH5α cells (ThermoFisher Scientific). For each LATS1 or CREBRF founder mouse, 48–96 bacterial colonies were picked for colony PCR, amplified, and sequenced individually after PCR product purification with LATS1 Fw Primer and CREBRF Fw Primer 2, respectively (Figure S1 and Figure S2). The CREBRF blastocysts were amplified using the primer sets reported in Table S1 and Sanger-sequenced directly.

### qPCR assays

The qPCR multiplex assays were performed on a StepOnePlus thermo cycler (ThermoFisher Scientific) using PerfeCTa MultiPlex qPCR ToughMix (Quanta Biosciences) or TaqMan Fast Advanced Master Mix (ThermoFisher Scientific), 30 μM of primer, 4.6 ng/reaction DNA, and 5 μM of reference and target probes in a 10 μl final reaction volume. For PerfeCta MultiPlex qPCR ToughMix, the PCR amplification profile was: one cycle of 50° for 2 min and 95° for 10 min; and 40 cycles of 95° for 15 sec and 60° for 1 min. Using TaqMan Fast Advanced Master Mix, the profile was: one cycle of 95° for 20 sec, and 40 cycles of 95° for 1 sec and 60° for 20 sec. For the *trans*-Multiplex Assay (ToughMix), the optimized PCR amplification profile was: one cycle of 50 for 2 min and 95° for 10 min; and 40 cycles of 95° for 15 sec, 59° for 1 min, and 60° for 1 min). All reactions were run in triplicate and the levels calculated using the ΔΔC_q_ method (Livak and Schmittgen 2001). To assess the compatibility of the probes used in each qPCR assay, a calibration curve was built and the ΔC_q_ slope approach was used.

### dPCR method and parameters

The dPCR analysis was performed on a QuantStudio 3D dPCR System using the manufacturer’s procedure and reagents (ThermoFisher Scientific). Data analysis and chip quality were assessed using the QuantStudio 3D Analysis Suite software online. Two different dilutions were tested to ensure that all chips were suitable and yielded reproducible quantification.

### Data analysis

Multiple sequence alignments were obtained using MultAlin Software (Corpet 1988). Statistical significance of the data was determined using the Student’s *t*-test in Microsoft Excel; all *P* values correspond to two-tailed two-sided sample *t*-test. The reported error bars represent the SEM. Pearson and Spearman correlation coefficients were calculated for 95% C.I.s using GraphPad Prism 7.

### Data availability

All primer sequences used are reported in Table S1. All primers were from IDT.

## Results

### Genetic complexity at the target site after CRISPR-Cas9 genome editing

**Figure 1:**
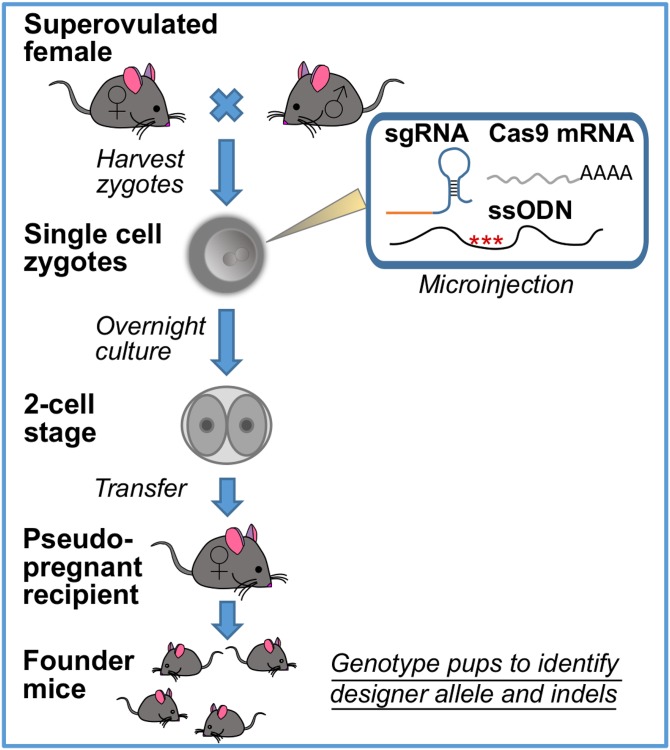
Workflow of CRISPR-Cas9-mediated production of founder mice. *In vitro*-fertilized oocytes from superovulated females generate single-cell zygotes, which are injected with the prepared sgRNA, Cas9 RNA, and ssODN and incubated overnight. The developed two-cell stage embryos are then implanted into a pseudopregnant female mouse to produce the founder mice, which are then characterized for CRISPR-Cas9 editing and bred to maintain the mouse line. CRISPR-Cas9, Clustered Regularly Interspaced Short Palindromic Repeats-CRISPR-associated protein 9; sgRNA, single guide RNA; ssODN, single-stranded oligodeoxynucleotide.

**Figure 2:**
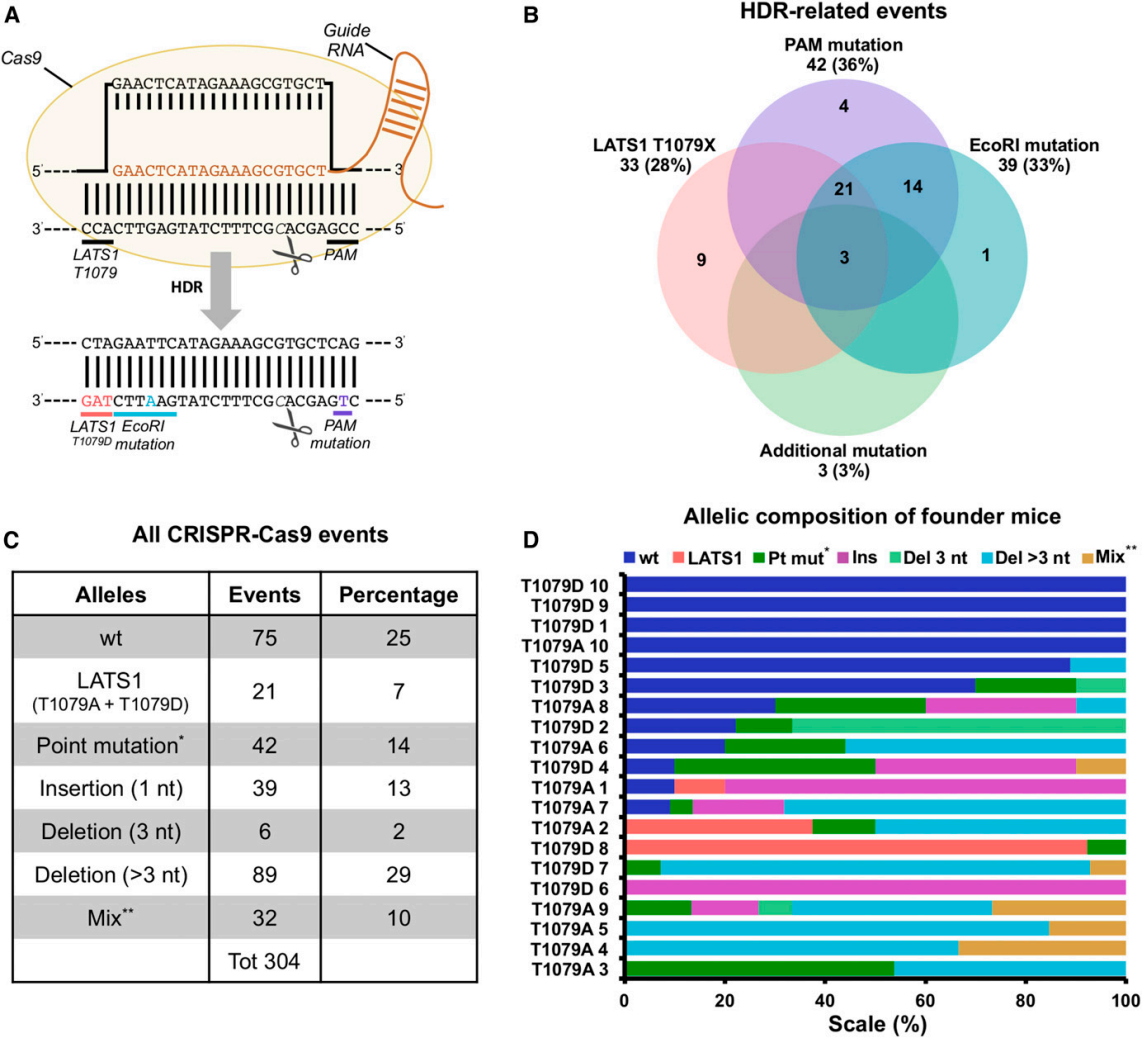
CRISPR-Cas9 genome editing creates significant allelic distribution in the LATS1 founder mice. (A) Diagram of the CRISPR-Cas9-mediated DNA cleavage and subsequent HDR to form LATS1 T1079D or T1079A mutation (data not shown). (B) Venn diagram showing the frequency of HDR-associated events based on the sequencing data of plasmid-cloned PCR products. (C) Distribution of 304 sequence variants by allele type. For this analysis, the sequence from GRCm38 chromosome 10: 7,712,787 to 7,712,856 nt was considered. (D) Allelic composition of 20 founder mice based on sequencing of PCR-clones. *inside or outside the selected type sequence; **ins/del, del/mut, mut/ins, or del/mut/ins (inside or outside the target sequence). CRISPR-Cas9, Clustered Regularly Interspaced Short Palindromic Repeats-CRISPR-associated protein 9; del, deletion; HDR, homology-directed repair; ins, insertion; LATS1, Large Tumor Suppressor Kinase 1; mut, mutation; nt, nucleotide; PAM, protospacer-adjacent motif; PCR, polymerase chain reaction.

To assess the genetic complexity derived from gene editing, we used a set of 20 founder mice generated by CRISPR-Cas9 mutagenesis (Figure 1) of the LATS1 gene. One 20 nt sgRNA was designed to target a unique genomic site on the murine LATS1 locus (Figure 2A). To generate HDR alleles, a ssODN was synthesized, carrying either the LATS1 T1079A or T1079D mutation, along with PAM mutations and *Eco*RI silent mutations (Figure 2A). To generate founder animals, sgRNA, Cas9 mRNA, and ssODN were microinjected into single-cell zygotes (Figure 1). After overnight culture, two-cell zygotes were transferred into pseudopregnant females and pups delivered at full term. At weaning, tail or toe snips were processed for total DNA extraction and a 476 bp PCR product generated. For each animal, the PCR product was cloned and ∼15 plasmid isolates per mouse were Sanger sequenced. PCR-cloned sequences allowed the determination of all variants on the same allele.

The hundreds of sequenced alleles provide insight into allelic complexity that can arise from *in vivo* CRISPR-Cas9 gene editing. Of 304 alleles analyzed, 21 contained the correct designed HDR (herein designated HDR), which is comprised of LATS1 (T1079A or T1079D), PAM, and *Eco*RI mutations. We also observed partial recombination occurring in 31 of 52 HDR-related sequences (Figure 2B), suggesting that incomplete HDR events can occur with high frequency and contribute to the genetic complexity induced by the CRISPR-Cas9 system. When examining HDR- and NHEJ-derived variants together, we observed numerous sequence types, which we categorized into unedited alleles (wt), LATS1 HDR mutation, deletions, point mutations, insertions, and mixed alleles (Figure 2C). Importantly, we noticed significant genetic mosaicism, ranging from homozygous to five different alleles within a founder DNA sample (Figure 2D). The occurrence of editing was not binary, with eight animals having variable abundance of wt alleles remaining. This degree of complexity would not be observed in cultured diploid cells, and would be difficult to quantify using T7 or surveyor methods. This observation motivated the development of faster gene editing quantification methods that can be planned concomitantly with sgRNA and ssODN design.

### Quantification of wt allele abundance in vivo after LATS1 CRISPR-Cas9 editing

**Figure 3:**
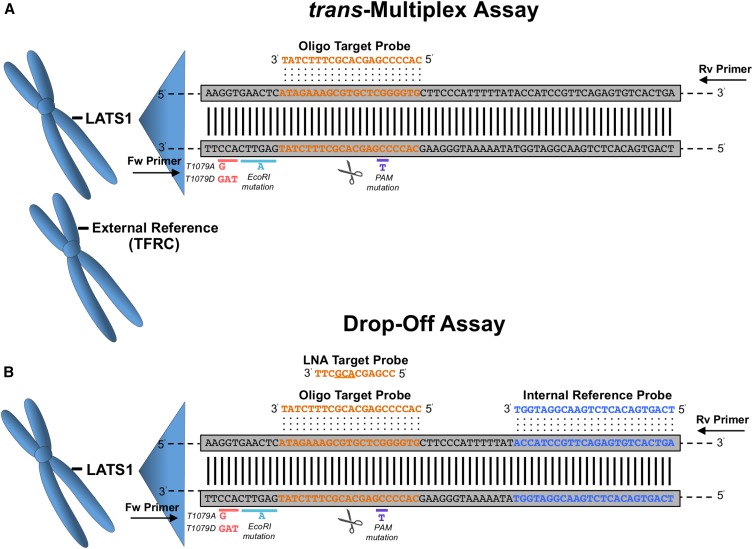
Schematic representation of three qPCR strategies for wt allele quantification after CRISPR-Cas9 editing in LATS1 mice. (A) *trans*-Multiplex Assay combines LATS1 Oligo Target qPCR with an External Reference qPCR (TFRC). The target probe (orange) binds at the Cas9 cleavage site (scissors). (B) Drop-Off Assay with both reference (blue) and target (orange) probes within the same amplicon. The LNA Target Probe (top) contains locked nucleotides (underlined), which increase detection specificity. Both Oligo and LNA Target Probes bind to wt sequence at the Cas9 cleavage site (scissors). CRISPR-Cas9, Clustered Regularly Interspaced Short Palindromic Repeats-CRISPR-associated protein 9; Fw, forward; LATS1, Large Tumor Suppressor Kinase 1; LNA, locked nucleic acid; PAM, protospacer-adjacent motif; qPCR, quantitative polymerase chain reaction; Rv, reverse; TFRC, Transferrin Receptor; wt, wild-type.

Prior work identified priming interference in singleplex SYBR qPCR assays as a valid approach to detect NHEJ editing (Yu *et al.* 2014). Rather than using PCR primer mismatch for detection as in that study, we used TaqMan probe mismatch for assessing mutations. For normalization, we used multiplex assays to reduce the number of reactions and the influence of pipetting error. We next designed and tested two different strategies, here named *trans*-Multiplex Assay and Drop-Off Assay (Figure 3). The difference between these assays lies in the normalization method. The *trans*-Multiplex Assay uses an External Reference Assay for the murine Transferrin Receptor (Figure 3A), while the Drop-Off Assay uses an Internal Reference Probe within the LATS1 amplicon yet distal to the mutation site (Figure 3B). We used the same amplification primers for the LATS1 region in all assays (amplicon size 134 bp). The Target probes base paired with the CRISPR-Cas9 guide sequence inclusive of the cleavage site. For this experiment, we tested both normal oligonucleotides (Oligo Target Probe) and LNAs (LNA Target Probe). The LNA Target Probe contains three locked nucleotides, connecting the 2’ oxygen and 4’ carbon within the same nucleotide, which increases the discrimination of mismatch sequences through improved specificity of probing when compared to an oligo probe (You *et al.* 2006).

**Figure 4:**
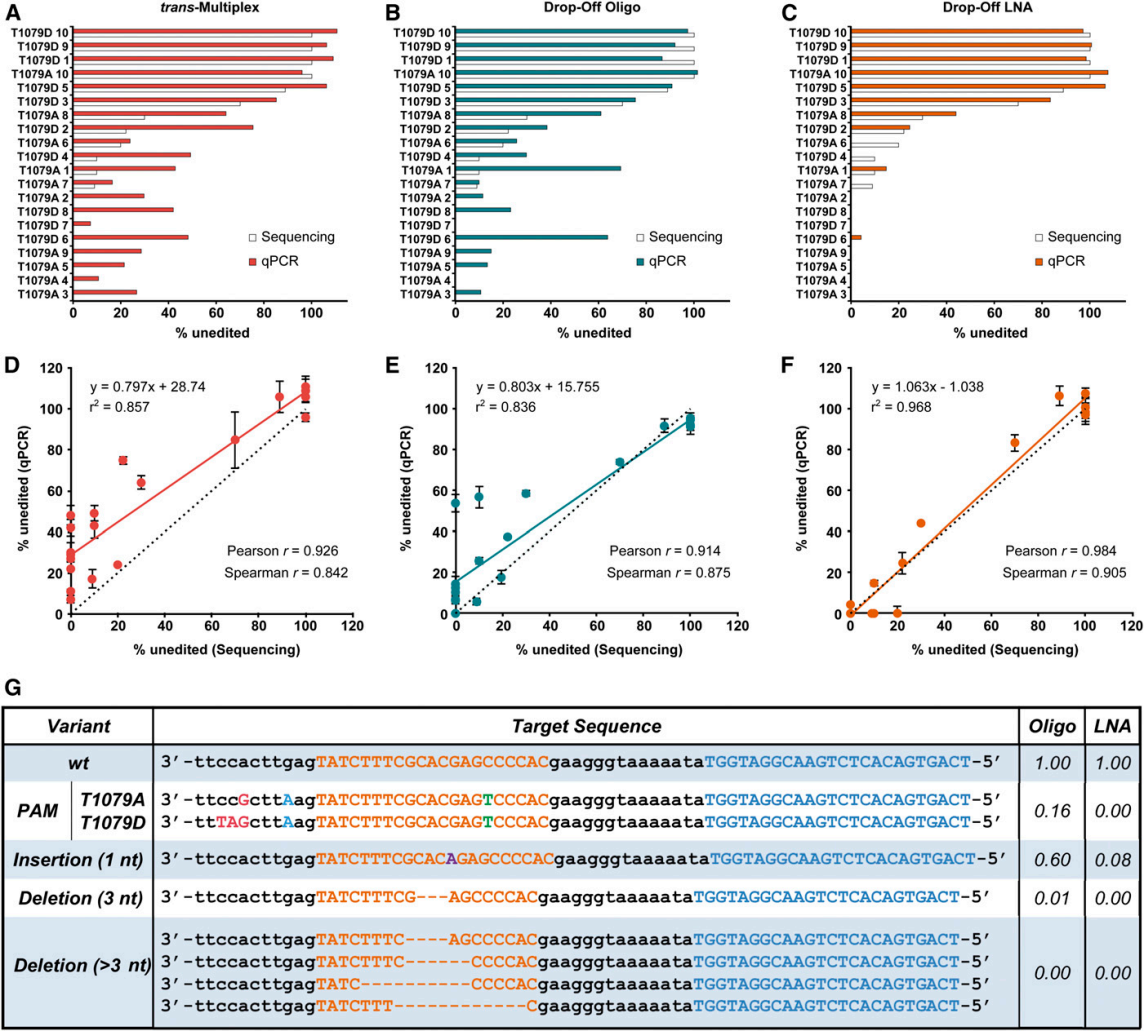
Drop-Off LNA Assay quantitatively detects CRISPR-Cas9 editing in LATS1 founder mice and shows higher sensitivity to editing on isolated target sequences. (A–C) Comparison between the extent of unedited alleles (wt) detected by sequencing (open bars) and qPCR analyses (filled bars) in individual founder transgenic mice. (D–F) Correlation of qPCR and sequencing data across founder animals using *trans*-Multiplex (A and D), Drop-Off Oligo (B and E), or Drop-Off LNA (C and F) Assays. The linear regression fit (solid line), ideal slope of 1.0 (dotted line), Pearson correlation coefficient, and Spearman correlation coefficient are shown for each assay. qPCR replicate data expressed as mean ± SEM (G) Amplification efficiency of Drop-Off Oligo and Drop-Off LNA qPCR Assays using templates that contain target sequence variants. Target (orange) and reference (blue) sequence regions are shown. CRISPR-Cas9, Clustered Regularly Interspaced Short Palindromic Repeats-CRISPR-associated protein 9; oligo, oligonucleotide; LATS1, Large Tumor Suppressor Kinase 1; LNA, locked nucleic acid; qPCR, quantitative polymerase chain reaction; wt, wild-type.

Next, we compared the extent to which the *trans*-Multiplex Assay, the Drop-Off Oligo Assay, or the Drop-off LNA Assays described the editing in the founder mouse DNA samples (Figure 4). For these qPCR analyses, we used the ∆∆C_q_ method (Ribot *et al.* 1998) and expressed the data as a percentage of unedited events (wt). Each assay was optimized and calibrated (Figure S1). The three qPCR assays detected unedited alleles in founder mice (Figure 4, A–C), although we observed a high rate of false-positive unedited events with both *trans*-Multiplex (Figure 4A) and Drop-Off Oligo Assays (Figure 4B) relative to the Sanger sequencing data. The underestimation of editing was more prominent in those mice characterized by a low percentage (< 8%) of wt as estimated by sequencing. To model the direct relationship between qPCR and sequencing data, linear regression analyses (Figure 4, D–F) showed that the Drop-Off LNA Assay (*r*^2^ = 0.968) produced the best correlation with Sanger sequencing data. The *trans*-Multiplex and Drop-Off Oligo Assays’ underestimation of editing was reflected by the numerous data points located above the idealized slope (Figure 4, D and E). To further describe the association between qPCR results and sequencing data, we performed Pearson linear correlation and Spearman rank-order correlation analyses on the results obtained from each method. The Drop-Off LNA Assay (Pearson *r* = 0.984 and Spearman *r* = 0.905) showed the highest degree of correlation relative to the *trans*-Multiplex Assay (Pearson *r* = 0.926 and Spearman *r* = 0.842) and Drop-Off Oligo Assay (Pearson *r* = 0.914 and Spearman *r* = 0.875). Importantly, the Drop-Off LNA Assay showed significantly higher sensitivity to all mutations and better accuracy for measuring editing when compared to the other two strategies. Our data highlight the utility of LNA-based over unmodified oligonucleotide-based methods for detecting and quantifying sequence mismatch.

To identify the differences in sensitivity between Oligo and LNA Target Probes, we tested the two Drop-Off Assays on representative isolated mutant target sequences. For this analysis, 14 target sequences from our Sanger sequencing data of the plasmid-clone PCR products were selected, including wt, PAM mutation, 1 nt insertion, 3 nt deletion, and > 3 nt deletion sequences (Figure 4G). We performed both Drop-Off Oligo and LNA qPCR Assays on variant and wt templates and expressed the amplification efficiencies relative to that of the wt sequence. Both Drop-Off methods detected deletions > 3 nt; however, the Drop-Off Oligo Assay moderately amplified both the PAM mutation and the 1 nt insertion sequences, indicating that this strategy is not sufficiently sensitive to detect all allelic variants and explaining why this assay underestimates editing in the founder mice.

### Combination of drop-off strategy with dPCR method to quantify on-target editing

**Figure 5:**
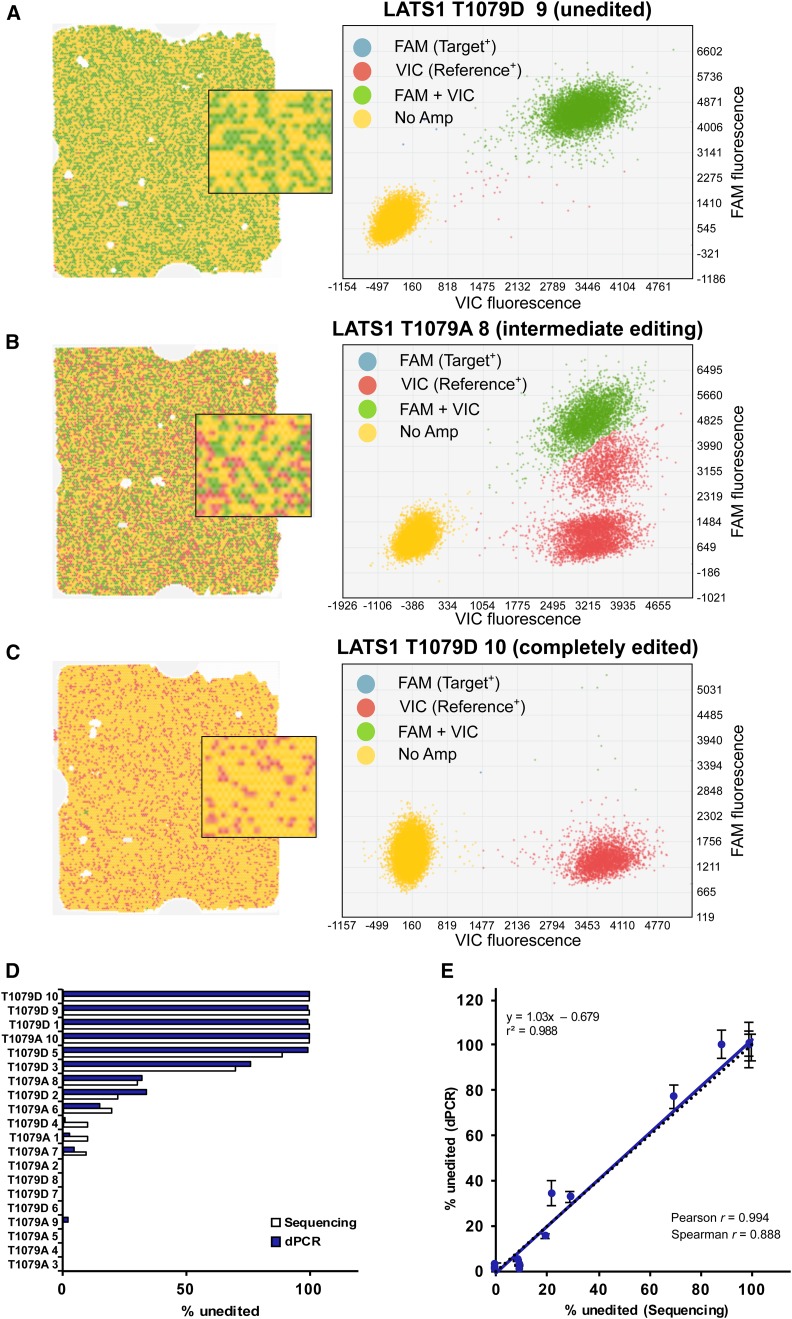
dPCR enhances the Drop-Off LNA Assay precision of detection of unedited alleles in founder mice. (A–C) Representative chips and scatter plots showing the distribution of Reference (red) and LNA Target (blue) probe signals in LATS1 founder mice samples that contain: (A) unedited DNA, (B) partially edited DNA, or (C) completely edited DNA. (D) Comparison of unedited alleles detected by sequencing (open bars) and dPCR (filled bars) analyses. (E) Correlation of Drop-Off LNA dPCR and sequencing data across founder animals. The linear regression fit (solid line), ideal slope of 1.0 (dotted line), Pearson correlation coefficient, and Spearman correlation coefficient are shown for each assay. dPCR replicate data expressed as mean ± SEM. Amp, amplification; dPCR, digital polymerase chain reaction; LATS1, Large Tumor Suppressor Kinase 1; LNA, locked nucleic acid.

qPCR assays, in general, show the strongest response in the midrange of the assay; as such, we observed decreased precision at low (< 15) and high (> 85) percent editing. Other technologies have improved sensitivity near these limits, such as dPCR. For most dPCR approaches, thousands to hundreds of thousands of single-molecule PCR reactions are performed to accurately measure the number of PCR-positive events independent of amplification efficiency. Using a chip-based dPCR system, we performed the Drop-Off LNA Assay on the 20 LATS1 founder mice (Figure 5). Example chip images (left) and scatterplot quantitation of probe results (right) showed clear differences in the extent of editing (Figure 5, A–C). Unedited samples were target and reference probe-positive (Figure 5A). Intermediate edited samples showed a mix of reference-positive and target-plus-reference-positive wells, and alleles with different amplification efficiency than wt (Figure 5B). Completely edited samples were only reference-positive (Figure 5C). We found that the dPCR Drop-Off LNA Assay improved measurement precision of unedited and highly edited DNA (Figure 5D), yielding slightly stronger correlation with the Sanger sequencing data (Figure 5E) compared to the qPCR data.

### Measuring CRISPR-Cas9 editing efficiency in blastocysts

**Figure 6:**
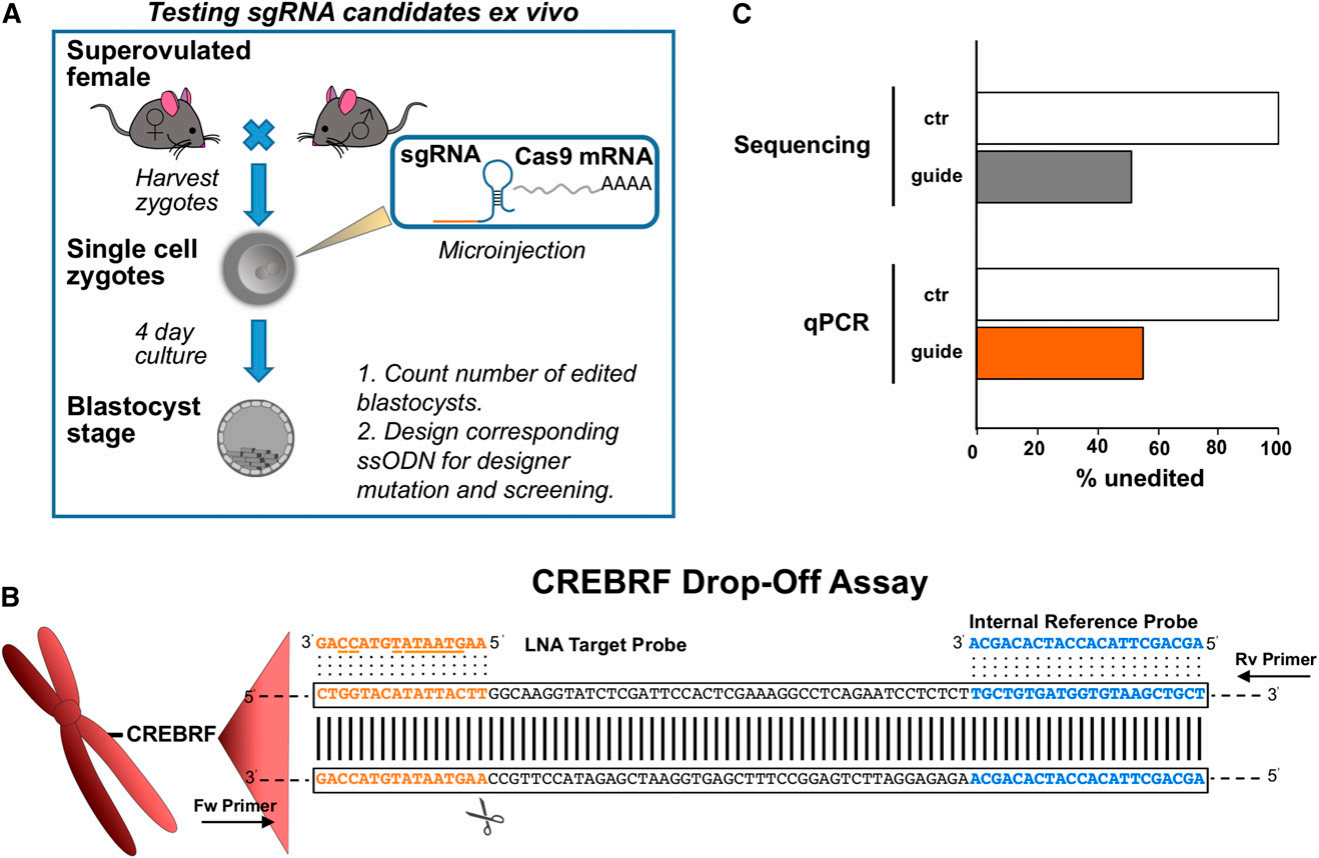
Drop-Off LNA qPCR Assay detects editing events in blastocysts. (A) Workflow for validating sgRNA guides in single-cell zygotes. (B) Schematic representation of the CREBRF Drop-Off LNA Assay and the CRISPR-Cas9 cleavage site (scissors). (C) Comparison of unedited events in ctr samples or sgRNA-injected blastocyst DNA (guide) as detected by sequencing and Drop-Off LNA qPCR analysis. CREBRF, CREB3 Regulatory Factor; CRISPR-Cas9, Clustered Regularly Interspaced Short Palindromic Repeats-CRISPR-associated protein 9; ctr, control DNA samples; Fw, forward; LNA, locked nucleic acid; qPCR, quantitative polymerase chain reaction; Rv, reverse; sgRNA, single guide RNA.

To measure editing efficiency without generating mice, injected single-cell embryos can be cultured until blastocyst stage *ex vivo* (Ran *et al.* 2013; Wang *et al.* 2013; Horii *et al.* 2014). The process is similar to that of transgenic mouse production; in this case, sgRNA and Cas9 mRNA are injected into single-cell embryos that are matured to blastocysts in culture (Figure 6A). Blastocysts are then harvested, DNA isolated, and the region of interest amplified for sequencing to detect editing. This procedure is effective for optimizing CRISPR-Cas9 reagents or selecting guide RNAs that will ultimately be used for transgenic mouse generation. Here, we tested whether a Drop-Off LNA Assay could be used for rapid qPCR detection of editing to substitute for sequencing. We designed a sgRNA guide that targeted the CREB3 Regulatory Factor (CREBRF) gene for NHEJ editing (Figure 6B). In parallel, a Drop-Off LNA Assay was developed and validated (Figure S2A) for detecting loss of wt amplification. We obtained 46 PCR products from 51 blastocysts, which were then subjected to direct Sanger sequencing and Drop-Off LNA qPCR (Figure 6C). The frequency of editing by Sanger sequencing and Drop-Off LNA qPCR was identical, extending the applicability of this method.

### Competitive qPCR with LNA probes to identify founder pups carrying recombinant alleles

**Figure 7:**
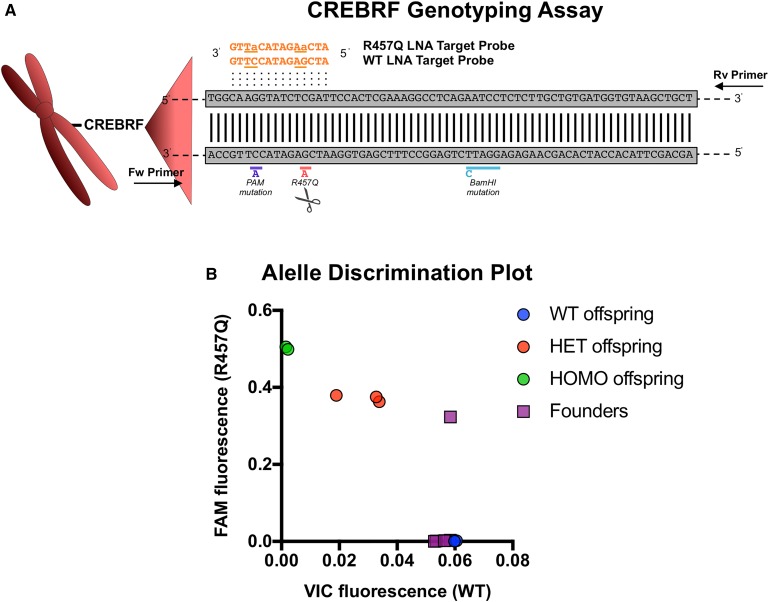
CREBRF genotyping assay using LNA Target Probes to detect the recombinant R457Q allele in founder mice. (A) Schematic representation of the allele competitive qPCR assay to detect CREBRF WT and R457Q sequences. The WT LNA and R457Q Target Probes (orange) contain locked nucleotides (underlined) at the PAM and R457Q mutation sites. (B) Allelic discrimination plot showing CREBRF founder mice (purple), and heterozygous (red), homozygous (green), and WT (blue) offspring controls that were validated by RFLP and sequencing analysis. CREBRF, CREB3 Regulatory Factor; HET, heterozygous; HOMO, homozygous; LNA, locked nucleic acid; PAM, protospacer-adjacent motif; qPCR, quantitative polymerase chain reaction; RFLP, restriction fragment length polymorphism; WT, wild-type.

The detection of recombinant alleles in founder mice is frequently built into the design of the donor sequence. A common approach includes silent mutations that add a restriction site within a diagnostic amplicon to generate a restriction fragment length polymorphism (RFLP), which can be used to demonstrate the presence of an allele after restriction and agarose gel electrophoresis. This PCR/RFLP approach is usually adequate for genotyping an established mouse line, where 0, 1, or 2 restriction-positive alleles can be easily distinguished. However, detection of low-abundance RFLP alleles in founder mice can prove to be challenging. To address recombinant allele detection in mosaic founder mice, we used an existing method for detecting single nucleotide polymorphisms: Competitive LNA PCR (Mouritzen *et al.* 2003). In contrast to the Drop-Off Assay, the fluorescent probes for WT or the CREBRF recombinant allele (R457Q) are designed for the same region and differentially labeled (Figure 7A). To detect the recombinant alleles, the terminal probe fluorescence is quantified for each sample and compared with appropriate controls in an allele discrimination plot (Figure 7B). To calibrate the assay, we used RFLP- and sequencing-validated wt, heterozygous, and homozygous offspring DNA samples and ensured linearity of dose response (Figure S2B). When the original six founder DNA samples were analyzed, the correct founder was identified. These data demonstrate the ability of LNA-based probes to rapidly identify the subset of edited mice carrying targeted mutant alleles after CRISPR-Cas9 editing.

## Discussion

We observed that CRISPR-Cas9-injected RNAs can generate broad allelic diversity even in the context of a donor ssODN recombination template. Importantly, we detected a range of alleles, from a homozygous single edit to five different edited variants in individual mice. Simultaneously, we also identified animals with no detectable CRISPR-Cas9 editing events. Although sgRNA, Cas9 RNA, and ssODN templates are injected into single-cell embryos, our data suggest that the observed *in vivo* allelic diversity may arise from target restriction occurring in later multicellular stages of development. Consistent with this notion, some delay would be expected due to translation, folding, and assembly of the sgRNA-Cas9 complex prior to endonuclease activity. Therefore, unlike in clonal populations of cells that carry at most two different alleles, measuring editing extent in founder animals benefits from a sensitive, quantitative method.

In this study, we introduced three important refinements in our PCR-based approach: the use of multiplex detection, the use of LNA probes, and dPCR. We did not observe significant differences in editing detection between the *trans*-Multiplex and Drop-Off Oligo approaches, which differ in the method of normalization, but we noted the convenience of an internal reference probe that is insensitive to differences in amplification efficiency between experimental and housekeeping targets. Detection of predicted or prescribed alleles by other methods, such as ligation-mediated or allele-specific PCR, may overlook editing events in founder lines that are potentially useful for gene disruption studies. Our data show that weak discrimination of some mutations can occur when standard oligonucleotides are used, but mutation detection is greatly improved with LNA probes. For dPCR, the limited quantitative differences between the qPCR and dPCR correlations with the PCR sequencing might suggest that the qPCR is sufficient for editing detection. However, because dPCR performs tens of thousands of reactions relative to the limited number of sequences per sample, we suggest that the correlation might be limited by the power of the sequencing data. For careful quantitation, or unambiguous exclusion of founders as unedited, we recommend dPCR.

In the current study, the internally referenced LNA probe system described here can be used as a founder generation screening tool, and based on our isolated allele data, would work for rapid genotyping of both recombinant and indel alleles in wt, heterozygous, and homozygous animals. While *cis*-sequencing remains necessary to describe the precise sequence of individual alleles produced by CRISPR-Cas9 editing *in vivo*, elimination of unedited founders and detection of sub-Mendelian levels of mutation will enable better colony management. The need for sequencing is further underscored by our detection of incomplete conversion of ssODN into targeted alleles, suggesting that even RFLP analysis is inadequate as a first-pass confirmation of HDR success.

In conclusion, our data demonstrate the high sensitivity of the Drop-Off LNA Assay for quantitative detection of CRISPR-Cas9-mediated gene editing. Based on the results presented herein, the Drop-Off Assay provides a rapid approach for initial identification and selection of high-fidelity HDR edits in founder mice.

## 

## Supplementary Material

Supplemental material is available online at www.g3journal.org/lookup/suppl/doi:10.1534/g3.117.300123/-/DC1.

## References

[bib1] Bassett A. Liu J.-L., 2014 CRISPR/Cas9 mediated genome engineering in Drosophila. Methods 69: 128–136. 24576617 10.1016/j.ymeth.2014.02.019

[bib2] Chen S. Sanjana N. E. Zheng K. Shalem O. Lee K., 2015 Genome-wide CRISPR screen in a mouse model of tumor growth and metastasis. Cell 160: 1246–1260. 25748654 10.1016/j.cell.2015.02.038 PMC4380877

[bib3] Corpet F., 1988 Multiple sequence alignment with hierarchical clustering. Nucleic Acids Res. 16: 10881–10890. 2849754 10.1093/nar/16.22.10881 PMC338945

[bib4] Cox D. B. T. Platt R. J. Zhang F., 2015 Therapeutic genome editing: prospects and challenges. Nat. Med. 21: 121–131. 25654603 10.1038/nm.3793 PMC4492683

[bib5] Dehairs J. Talebi A. Cherifi Y. Swinnen J. V., 2016 CRISP-ID: decoding CRISPR mediated indels by Sanger sequencing. Sci. Rep. 6: 28973. 27363488 10.1038/srep28973 PMC4929496

[bib6] Heidenreich M. Zhang F., 2016 Applications of CRISPR-Cas systems in neuroscience. Nat. Rev. Neurosci. 17: 36–44. 26656253 10.1038/nrn.2015.2 PMC4899966

[bib7] Horii T. Arai Y. Yamazaki M. Morita S. Kimura M., 2014 Validation of microinjection methods for generating knockout mice by CRISPR/Cas-mediated genome engineering. Sci. Rep. 4: 4513. 24675426 10.1038/srep04513 PMC5380110

[bib8] Jinek M. Chylinski K. Fonfara I. Hauer M. Doudna J. A., 2012 A programmable dual-RNA-guided DNA endonuclease in adaptive bacterial immunity. Science 337: 816–821. 22745249 10.1126/science.1225829 PMC6286148

[bib9] Kc R. Srivastava A. Wilkowski J. M. Richter C. E. Shavit J. A., 2016 Detection of nucleotide-specific CRISPR/Cas9 modified alleles using multiplex ligation detection. Sci. Rep. 6: 1–7. 27557703 10.1038/srep32048 PMC4997339

[bib10] Kolesar J. E. Wang C. Y. Taguchi Y. V. Chou S.-H. Kaufman B. A., 2013 Two-dimensional intact mitochondrial DNA agarose electrophoresis reveals the structural complexity of the mammalian mitochondrial genome. Nucleic Acids Res. 41: e58. 23275548 10.1093/nar/gks1324 PMC3575812

[bib11] Livak K. J. Schmittgen T. D., 2001 Analysis of relative gene expression data using real-time quantitative PCR and. Methods 25: 402–408. 11846609 10.1006/meth.2001.1262

[bib12] Mali P. Esvelt K. M. Church G. M., 2013 Cas9 as a versatile tool for engineering biology. Nat. Methods 10: 957–963. 24076990 10.1038/nmeth.2649 PMC4051438

[bib13] Mouritzen P. Nielsen A. T. Pfundheller H. M. Choleva Y. Kongsbak L., 2003 Single nucleotide polymorphism genotyping using locked nucleic acid (LNA). Expert Rev. Mol. Diagn. 3: 27–38. 12528362 10.1586/14737159.3.1.27

[bib14] Oliver D. Yuan S. McSwiggin H. Yan W., 2015 Pervasive genotypic mosaicism in founder mice derived from genome editing through pronuclear injection. PLoS One 10: e0129457. 26053263 10.1371/journal.pone.0129457 PMC4459985

[bib15] Pelletier S. Gingras S. Green D. R., 2015 Mouse genome engineering via CRISPR-Cas9 for study of immune function. Immunity 42: 18–27. 25607456 10.1016/j.immuni.2015.01.004 PMC4720985

[bib16] Ran F. A. Hsu P. D. Lin C. Y. Gootenberg J. S. Konermann S., 2013 Double nicking by RNA-guided CRISPR cas9 for enhanced genome editing specificity. Cell 154: 1380–1389. 23992846 10.1016/j.cell.2013.08.021 PMC3856256

[bib17] Ribot E. M. Quinn F. D. Bai X. Murtagh J. J., 1998 Comparative PCR: an improved method to detect gene amplification. Biotechniques 24: 22–26. 9454944 10.2144/98241bm02

[bib18] Ruiz S. Mayor-Ruiz C. Lafarga V. Murga M. Vega-Sendino M., 2016 A genome-wide CRISPR screen identifies CDC25A as a determinant of sensitivity to ATR inhibitors. Mol. Cell 62: 307–313. 27067599 10.1016/j.molcel.2016.03.006 PMC5029544

[bib19] Sternberg S. H. Doudna J. A., 2015 Expanding the biologist’s toolkit with CRISPR-Cas9. Mol. Cell 58: 568–574. 26000842 10.1016/j.molcel.2015.02.032

[bib20] Wang H. Yang H. Shivalila C. S. Dawlaty M. M. Cheng A. W., 2013 One-step generation of mice carrying mutations in multiple genes by CRISPR/Cas-mediated genome engineering. Cell 153: 910–918. 23643243 10.1016/j.cell.2013.04.025 PMC3969854

[bib21] Yang Z. Steentoft C. Hauge C. Hansen L. Thomsen A. L., 2015 Fast and sensitive detection of indels induced by precise gene targeting. Nucleic Acids Res. 43: e59. 25753669 10.1093/nar/gkv126 PMC4482057

[bib22] You Y. Moreira B. G. Behlke M. A. Owczarzy R., 2006 Design of LNA probes that improve mismatch discrimination. Nucleic Acids Res. 34: e60. 16670427 10.1093/nar/gkl175 PMC1456327

[bib23] Yu C. Zhang Y. Yao S. Wei Y., 2014 A PCR based protocol for detecting indel mutations induced by TALENs and CRISPR/Cas9 in zebrafish PLoS One 9: e98282. 24901507 10.1371/journal.pone.0098282 PMC4046980

[bib24] Zentilin L. Giacca M., 2007 Competitive PCR for precise nucleic acid quantification. Nat. Protoc. 2: 2092–2104. 17853864 10.1038/nprot.2007.299

[bib25] Zhang X. Liang P. Ding C. Zhang Z. Zhou J., 2016 Efficient production of gene-modified mice using Staphylococcus aureus Cas9. Sci. Rep. 6: 32565. 27586692 10.1038/srep32565 PMC5009317

